# A Model of Implant-Associated Infection in the Tibial Metaphysis of Rats

**DOI:** 10.1155/2013/481975

**Published:** 2013-12-08

**Authors:** Maximilian Haenle, Carmen Zietz, Tobias Lindner, Kathleen Arndt, Anika Vetter, Wolfram Mittelmeier, Andreas Podbielski, Rainer Bader

**Affiliations:** ^1^Department of Orthopaedics, University Medicine Rostock, Doberaner Straße 142, 18057 Rostock, Germany; ^2^Institute of Medical Microbiology, Virology and Hygiene, University Rostock, Schillingallee 70, 18057 Rostock, Germany

## Abstract

*Objective*. Implant-associated infections remain serious complications in orthopaedic and trauma surgery. A main scientific focus has thus been drawn to the development of anti-infective implant coatings. Animal models of implant-associated infections are considered helpful in the *in vivo* testing of new anti-infective implant coatings. The aim of the present study was to evaluate a novel animal model for generation of implant-associated infections in the tibial metaphysis of rats. *Materials and Methods*. A custom-made conical implant made of Ti6Al4V was inserted bilaterally at the medial proximal tibia of 26 female Sprague-Dawley rats. *Staphylococcus aureus* in amounts spanning four orders of magnitude and each suspended in 15 **μ**l phosphate buffered saline (PBS) was inoculated into the inner cavity of the implant after the implantation into the defined position. Controls were treated accordingly with PBS alone. Animals were then followed for six weeks until sacrifice. Implant-associated infection was evaluated by microbiological investigation using swabs and determination of viable bacteria in the bone around the implant and the biofilm on the implants after sonification. *Results*. Irrespective of the initial inoculum, all animals in the various groups harbored viable bacteria in the intraoperative swabs as well as the sonication fluid of the implant and the bone samples. No correlation could be established between initially inoculated CFU and population sizes on implant surfaces at sacrifice. However, a significantly higher viable count was observed from peri-implant bone samples for animals inoculated with 10^6^ CFU. Macroscopic signs of animal infection (pus and abscess formation) were only observed for implants inoculated with at least 10^5^ CFU *S. aureus*. *Discussion/Conclusion*. The results demonstrate the feasibility of this novel animal model to induce an implant-associated infection in the metaphysis of rats, even with comparatively low bacterial inocula. The specific design of the implant allows an application of bacteria in reproducible numbers at well-defined contact sites to the animal bone.

## 1. Introduction

Implant-associated infections remain feared and severe complications in orthopaedic and trauma surgery. Beside the vast pathological and psychosocial significance for the patient, an enormous economic impact can be observed for the hospital in charge and consequently the healthcare system [1–4]. Despite strict specific hygiene measures such as peri-operative antibiotic prophylaxis, laminar-air flow operation theatres and the use of sterishields, implant-associated infection rates remained more or less consistent throughout the past two decades [4]. The frequency of infection however is increasing with an increasing number of total joint arthroplasties [4, 5].

Treatment of implant-associated infections is complicated and often of small success because of the special biofilm growth characteristics of the responsible bacteria. First, biofilm growth protects bacteria from host defense mechanisms. Second, biofilm forms a diffusion barrier against systemically applied antibiotics [6–8], leading to a decreased susceptibility towards such antibiotics [9–11]. The problem could be aggravated when multiresistant pathogens such as Methicillin-resistant *Staphylococcus aureus *(MRSA), vancomycin-resistant *Enterococci* (VRE), or multi-resistant Gram-negative rods cause the infections [12–15].

In this context, anti-infective implant coatings could display dual advantage both in prevention and treatment of implant-associated infections. Therefore, the development of such anti-infective implant coatings has become a major scientific focus. Yet, despite many promising approaches no final breakthrough has been achieved in clinical practice [16, 17].

Animal models of implant-associated infections are considered helpful for *in vivo *testing of potential anti-infective implant coatings and antibiotics. In addition, these models could improve the understanding of the specific pathogenesis as well as could support the optimization of surgical techniques. Consequently, a number of different models of implant-associated infections have been developed [18–22]. The models themselves and individual adjuvants such as soft tissue manipulation are still under discussion [19, 21]. Among other crucial parameters the amount of seeding bacteria is subject to controversial debates. To promote signs of infection, bacterial counts ranging from as little as 10^2^ CFU of *S. aureus* [21] to 10^6^ CFU of *S. aureus* have been used [20]. Another moot point is the seeding time point in respect to implant insertion, that is, should the implants be covered by a preformed biofilm or should the bacteria be applied during surgery, which would closely mimic the natural situation.

Hence the aim of the present study was to evaluate a novel animal model for generation of implant-associated infections in the tibia metaphysis, in which the amount of bacterial inocula necessary for generating an infection was tested as an independent parameter.

## 2. Materials and Methods

### 2.1. Bacteria Strains and Preparation of Inocula

*Staphylococcus aureus* (ATCC 25923) was used for this study as a pathogen of implant-associated infection. The strain was grown in Caso-Bouillon (CB) (Carl Roth, Karlsruhe, Germany) in an overnight culture at 37°C under a 5% CO_2_–20% O_2_ atmosphere. Bacterial counts as displayed in Table 1 were obtained by washing and suspending these cultures in PBS (2 g KCl, 2.4 g KH_2_PO_4_, 80 g NaCl, and 14.4 g Na_2_HPO_4_ per 1000 ml; pH 7.4) and spectrophotometric control (Smart Spec 3000, Bio-Rad Laboratories GmbH, München, Germany).

For preparation of 1 ml deep frozen stocks, 10% glycerol was added to the defined concentrations. The stock suspensions were kept at −70°C until the day of surgery. To quantify a possible loss of viable bacteria during deep-freezing, bacterial CFU were counted from 15 *μ*l aliquots 24 hours after deep freezing and in parallel to each animal experiment. Viability counts were performed by serial tenfold dilution of the initial 15 *μ*l aliquots in PBS. From each dilution step, a 100 *μ*l aliquot was transferred onto Columbia sheep blood agar (Becton-Dickinson, Heidelberg, Germany). The strain identity was determined to species level in each experiment by judging colony morphology and performing catalase and coagulase tests. For identification to strain level, the *spa* gene of the isolate was amplified by PCR and the resulting PCR product was sequenced. The obtained *spa* gene sequence was compared to a commercial database. Strains belonging to the *spa* type of *S. aureus* ATCC25923 were regarded as identical with the inoculum strain.

### 2.2. Animals and Surgical Procedure

Female Sprague-Dawley rats (Charles River Laboratories, Germany GmbH, Sulzfeld, Germany) were used for this study. Animal experiments have been approved by the local review committee of the Landesamt für Landwirtschaft, Lebensmittelsicherheit und Fischerei M-V (LALLF MV, Reference number 7221.3-1.1-031/09). For acclimatization, the animals were delivered to the animal facility at least one week prior to first treatment. Animals were housed in cages at normal room temperature and daylight illumination with free access to food and water. They were treated according to current guidelines on animal well-being as previously approved by the Local Committee for Animal Experimentation (Reference number 7221.3-1.1-031/09).

Animals were randomly selected for each group. Surgery was performed under general anesthesia. This was induced by an intramuscular injection of 150 *μ*g/kg medetomidine (Dorbene vet, Fort Dodge, Würselen, Germany), 200 *μ*g/kg Midazolam (Ratiopharm, Ulm, Germany), and 5 *μ*g/kg Fentanyl (Ratiopharm). The medial metaphysis of both tibiae was chosen as surgical site.

**Figure 1:**
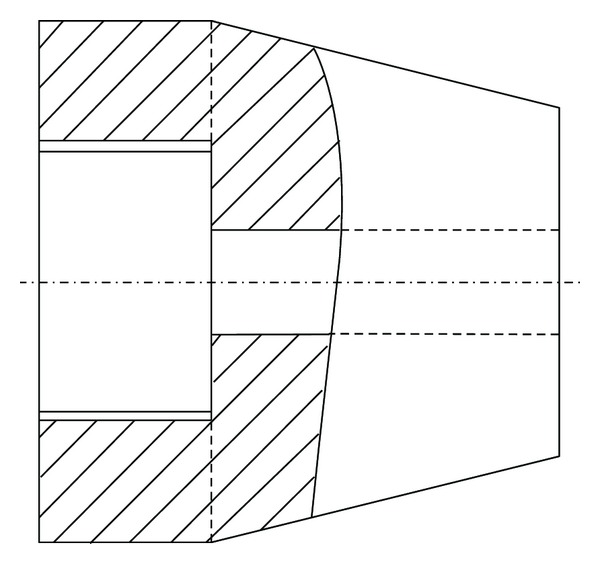
Schematic drawing of the canulated implant (diameter = 3 mm; length = 3 mm).

**Figure 2:**
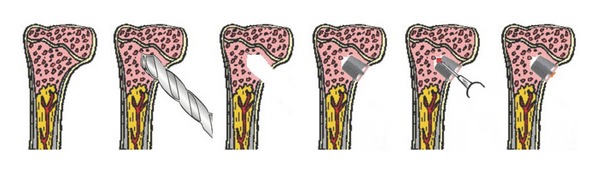
Schematic presentation of the surgical procedure. Cartoons 1 to 3: preparation of the implant bed, cartoon 4: implantation, cartoon 5: inoculation of bacteria into the implant cavity, and cartoon 6: wax sealing of the implant cavity.

The skin at the surgical site was shaved and disinfected with octenidine hydrochloride plus phenoxyethanol (Octenisept, Schülke & Mayr, Norderstedt, Germany) before sterile draping. A medial incision of skin and fascia was performed in sterile surgical technique. The preparation of the implant bed was performed using a circular drill (2.8 mm diameter). A custom-made conical implant (3 mm maximum outer diameter and 3 mm length) made of Ti6Al4V was implanted bilaterally into the rats' medial tibia metaphysis (Figures 1 and 2) followed by the injection of 15 *μ*l PBS containing *Staphylococcus aureus* into the implant cavity using a 25 *μ*l microsyringe (Hamilton, Reno, NV). Four different bacterial counts (10^6^ CFU, 10^5^ CFU, 10^4^  CFU, and 10^3^ CFU) of *Staphylococcus aureus *were used in the different experiments for seeding of the peri-implant bone via the hollow implant. Controls received identical amounts of sterile PBS into identical types of implants. The outer opening of the implant cavity was finally sealed with bone wax.

Surgical sites were closed using Vicryl sutures (Ethicon, Somerville, NJ, USA). After the operation anesthesia was antagonized by an intramuscular injection of 750 *μ*g/kg atipamezole (Alzane, Pfizer, Berlin, Germany), 200 *μ*g/kg Flumazenil (Flumazenil-ratiopharm, Ratiopharm, Ulm, Germany), and 120 *μ*g/kg naloxone (Naloxon-Ratiopharm, Ratiopharm, Ulm, Germany). When necessary, 50 *μ*g/kg buprenorphine (Temgesic, Essex Pharma, München, Germany) was injected intramuscularly as postoperative analgesic.

A total of 26 animals were then followed for six weeks until sacrifice. Body temperature and weight were weekly determined. Blood samples were collected prior to sacrifice in order to analyze red blood cell count, white blood cell count, and C-reactive protein levels. Radiographs of the tibial bone were prepared immediately after surgery, three weeks after surgery, and prior to the explantation.

The animals were sacrificed after 6 weeks under general anesthesia, induced as previously described with an overdose of pentobarbital 80 mg/kg. Surgical sites were again shaved and disinfected with octenidine hydrochloride plus phenoxyethanol combination (Octenisept, Schülke & Mayr) before sterile draping. Employing sterile surgery techniques, skin incision within the old scar was performed. Initially, swabs (AMIES W/O CH, Sarstedt, Nümbrecht, Germany) were premoistened with sterile saline and streaked into the wounds of each animal once tibia and implant became visible. During the procedure, direct skin contact was carefully avoided to minimize the risk of contamination. Tibiae were then excised under sterile conditions. Bones and implants were finally separated using surgical instruments and stored in sterile PBS (10 ml for tibia specimens; 1 ml for implants).

In total, 6 specimens from Group I (Control), 10 specimens from Group II, 16 specimens from Group III, 16 specimens from Group IV, and finally 4 specimen from Group V were assigned to microbiological examination. Two specimens of Group I were histologically examined.

### 2.3. Examination of Bacteriology Swabs

Bacteriology swabs obtained as described above were moistened with one drop of sterile PBS, then evenly streaked onto a plate each of Columbia blood agar, Schaedler agar, and MacConkey agar, and thereafter immersed in nutrient broth and brain-heart infusion. The solid and liquid media were incubated at the below mentioned conditions:

 Columbia blood agar (BD): 37°C + 5% CO_2_; 24 h,
 MacConkey agar (BD): 37°C + 5% CO_2_; 48 h,
 Schaedler agar (BD): 37°C under anaerobic conditions; 24 h,
 Nutrient Broth 1 (Neogen, Lansing MI, USA): 37°C + 5% CO_2_; 24 h,
 BHI medium (BD): 37°C under anaerobic conditions; 24 h.

After 24 h or 48 h, respectively, the solid and liquid media were analyzed by conventional bacteriology techniques. The identity of potential *S. aureus* isolates was determined to the species level by mass spectrometry (Vitek Mass Spectrometer, BioMerieux) and to strain level by *spa* typing.

### 2.4. Microbiological Examination of the Implant

Implant-adhering bacteria were detached from the implant immersed in 1 ml PBS using low frequency ultrasound treatment (Sonorex digital 10P, Bandelin, Berlin, Germany: 5 min. at 80% intensity) [23, 24]. After the treatment, tubes were centrifuged at 4000 rpm for 10 min. at 4°C temperature (Heraeus Varifuge 3.OR; Kendro Laboratory Products, Osterode, Germany). The supernatant was resuspended in 300 *μ*l PBS. Viable bacteria were determined as described above.

### 2.5. Microbiological Examination of Peri-Implant Bone

Cleaned tibia specimens in 10 ml PBS were exposed for 10 sec. to vigorous shaking (Vortex Genie 1, Scientific Industries, USA). Then the tibiae were removed from the resulting suspension and separately analyzed at a later stage.

The suspension was centrifuged at 4000 rpm for 10 min at 4°C temperature (Heraeus Varifuge 3.OR; Kendro Laboratory Products, Osterode, Germany). The sediment was resuspended in 3 ml PBS. For quantitative assessment of viable bacteria, 1 ml aliquots were serially diluted. One hundred *μ*l aliquots from each dilution step were plated on Columbia blood agar plates and incubated for 24 h at 37°C under a 5% CO_2_/20% O_2_ atmosphere. The bone was weighed in a sterile petri dish, crushed, and prepared for DNA isolation and real-time polymerase chain reaction (PCR) as described above.

### 2.6. Statistics

Quantitative data is displayed as mean ± standard deviation (SD). Initially the Kruskal-Wallis test as a one-sided analysis of variance was applied. Where applicable, the Mann-Whitney test for independent samples and the Wilcoxon test for dependent samples were used for the statistical analysis. For all tests, the level of significance was set to *P* < 0.05.

## 3. Results

### 3.1. X-Ray Investigation

**Figure 3:**
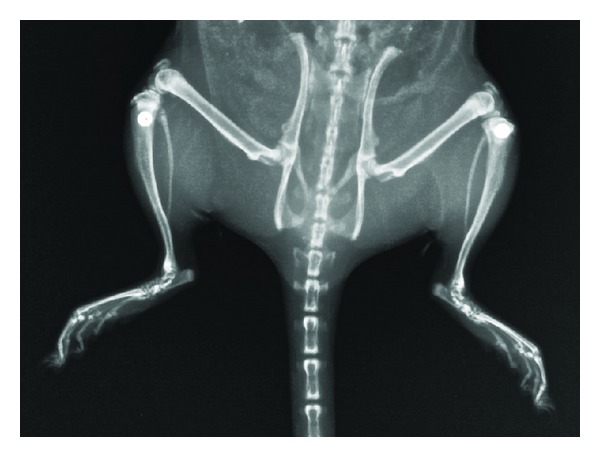
X-ray examination after surgery with the implant properly positioned in the proximal tibial metaphysis.

Neither in one of the *S. aureus*-exposed Groups II–V nor in the sterile control Group I were radiographic signs of infection present three weeks post-operatively and prior to sacrifice (Figure 3).

### 3.2. Body Weight and Temperature

Animals from Groups I–V showed an initial decrease in body weight postoperatively without any statistical significance within or between the groups (*P* > 0.05). Furthermore, a gain in body weight was observed in animals from Groups I–V prior to sacrifice. This again was without any statistical significance within the groups (*P* > 0.05).

No obvious differences in rectal temperature were found between Groups I–V at any time of animal examination.

### 3.3. Blood Cell Count and CRP

Blood samples taken at the time of sacrifice revealed no differences of red blood cell count (RBC) or white blood cell count (WBC) within Groups I–V (*P* > 0.05). Furthermore, no statistical differences were found regarding the CRP values between the groups (*P* > 0.05) (Table 2).

### 3.4. Macroscopic Evaluation

In 3 animals of Group V (10^6^ CFU) and 1 animal of Group IV (10^5^ CFU) macroscopic pus and abscess formation was observed. Implant dislocation was observed in one animal from Group V as well as in one animal from Group II. Neither signs of implant dislocation nor pus and abscess formation was found for the remaining animals of all Groups I–V.

### 3.5. Microbiological Investigation

**Figure 4:**
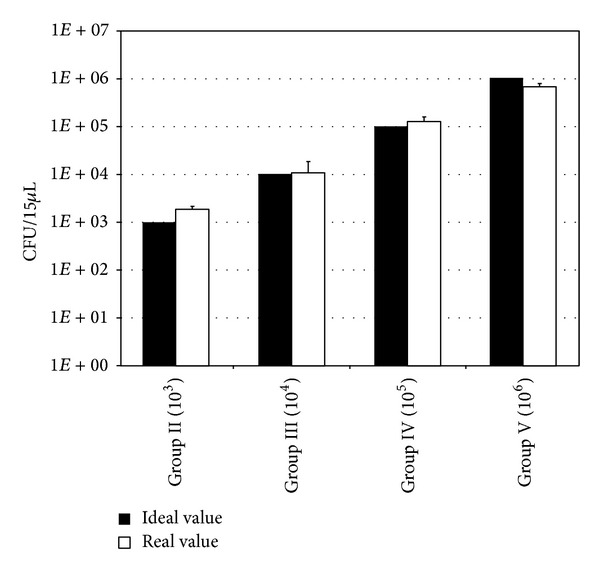
Ideal and experimentally determined CFU values of *S. aureus* bacteria prior to inoculation.

Average viable counts of bacteria recovered from samples of the *S. aureus*-exposed animal groups are summarized in Table 1 and Figure 4.

After sacrifice of animals of Groups II to V, *S. aureus *was isolated from all bacteriology swabs, as well as from the corresponding sonification fluid of the implant and the peri-implant bone samples. The isolates were identified to species level as *S. aureus *and to strain level as ATCC 25923 as described in Section 2. In all cases, the animal isolates corresponded to the *S. aureus* strain inoculated during initial surgery. In contrary, specimens taken from the PBS controls (Group I) remained sterile.

**Figure 5:**
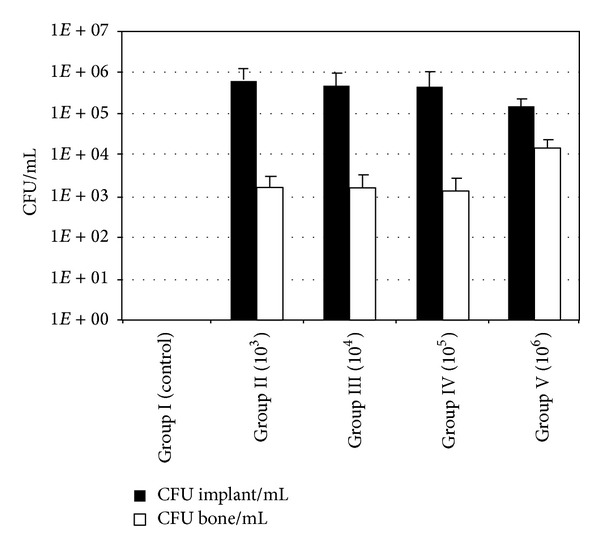
Bacterial counts cultured from the implant sonication fluids and suspensions prepared from periprosthetic tibial metaphysis.

When analyzing the quantitative data from microbiological investigation of the sonication fluid and the peri-implant bone specimens, the *S. aureus*-exposed Groups II–V displayed numbers of viable bacteria well above the baseline defined by the control Group I treated with sterile fluids (Figure 5; Table 3). Furthermore, comparison of the bacterial quantities recovered from the implant-derived sonication fluid revealed no statistical significant (*P* > 0.05) differences between Groups II–V (Figure 5; Table 3). Moreover, quantitative microbiological investigation from the bone revealed no statistical difference in CFU/ml between Groups II–IV (*P* > 0.05) (Figure 5; Table 3), whereas a significant difference of CFU/ml from the peri-implant bone was observed between Group V and Groups II–IV (*P* < 0.05) (Figure 5; Table 3).

## 4. Discussion

With a constantly rising demand for orthopaedic and trauma surgery, the frequency of associated infections is bound to increase. Studies on implant-associated infections and potential anti-infective implant coating involving animal models are therefore urgently needed.

Most models of implant-associated infection in rats, mice, and rabbits are using intramedullary implants [20–22, 25] or plates [18, 26] and therefore mimic situations from trauma surgery. To our best knowledge, no animal model of implant-related-associated infection of the metaphyseal bone has so far been established.

Therefore, we intended to develop a more precise model for the generation of an implant-associated infection for such implants which also includes the option of extended implantation periods covering the complete “early infection” period up to two months after implantation [27]. Consistently, we chose a comparatively long observation period of six weeks in the present study.

With this study, we also addressed a crucial but moot point: the amount of bacteria to be used in animal models of infected implants is still controversially discussed. Monzón et al. [20] demonstrated that only 25% of animals with sterile tibia implants revealed signs of infection when exposed to a suspension of 10^5^ CFU *S. aureus*. Usage of implants precolonized by a bacterial biofilm and additionally the administration of a suspension with 10^6^ CFU *S. aureus* was therefore regarded as a reliable model to produce implant-related infections [20].

On the other hand, Lucke et al. [21] developed a model of implant-associated osteomyelitis in rats, displaying histological, microbiological, and radiological signs of infection with as little as 10^3^ CFU of *S. aureus *[21]. In the present study, we were able to establish constant bacterial presence on the implant and in its environment and could demonstrate growth to common steady state value irrespective of the initial inocula, which differed by 3 orders of magnitude. However, only with large numbers of bacteria classical signs of local inflammation could be induced. From all bacteriology swabs, implant sonication fluids, and peri-implant bone samples, *S. aureus* (ATCC 25923) was detectable. Thus, we were able to support the findings of Lucke et al. [21] who induced an implant-associated infection with as little as 10^3^ CFU of *S. aureus. *Thus we were able to abdicate on further histological investigation of the implants' as microbiological proof of viable bacteria was evident in all septic samples. Histological investigation was therefore mainly performed in a descriptive manor in order to rule out any adverse reaction.

It has previously been argued that the choice of tested implants requiring large bacterial inocula to generate implant-related osteomyelitis [20] could lead to the discrepancies observed between former studies [21]. Hollow implants were thereby hypothesized to provide a contaminated space not accessible for host defence mechanisms [21]. On the other hand it is an accepted statement in clinical practice that implant-associated infection in orthopaedic and trauma surgery could be related to low bacterial inocula even for species of low virulence [27–29]. Another controversial point is bone quality, since rat bone structure and metabolism are different from that of human bones [30], which for itself could be a reason for diverse minimum bacterial inocula necessary in different test settings.

**Figure 6:**
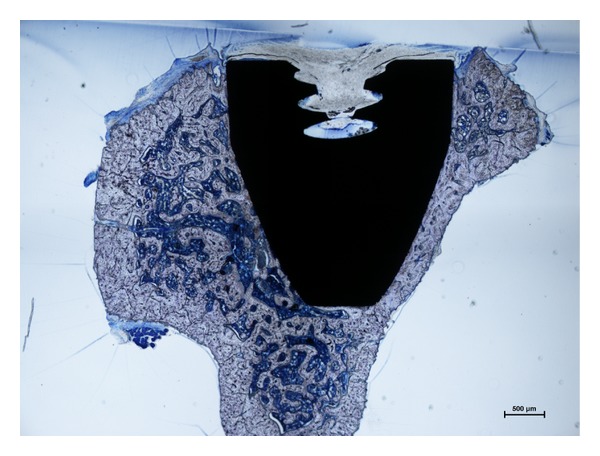
Histological cross-section of the tibial metaphysis including the inserted implant from an animal of Group I (uninfected control). The animal was sacrificed 42 d after the surgical implantation. The cross section shows no microscopic sings of infection and tissue ingrowth into the cavitated part of the implant.

In our novel model of implant-associated infection the implant bears a relatively small, cannulated space. With respect to a “dead space” hypothesis, this space however is considered negligible since the distal part of the cannula is in constant contact with the bone marrow. Hence the space is at least partially accessible to host defence mechanisms. Histological cross-sectional cuts of the control tibia furthermore proofed tissue in growth, at least into the threaded part of the implant (Figure 6). Moreover, this model design mimics the thread of many well-established total joint systems of numerous manufacturers. Nevertheless, for certain study protocols, such as the investigation of potential anti-infectious implant coatings, this might be considered a limitation. This is due to the fact that certain implant coatings may not be applied to all implant shapes and cavities with small diameter. Furthermore, despite introducing an implant-related infection in the tibial metaphysis of rats, the knee joint was not opened.

In contrast to previous studies [21], we found no quantitative correlation between the amount of bacteria initially inoculated during surgery and viable bacteria retrieved from peri-implant bone (Figure 5; Table 3). Only in the bone material from Group V (initial inoculum 10^6^ bacteria) a significantly (*P* < 0.05) higher count of viable bacteria was found compared to the bone material from Groups II to IV (initial inocula 10^3^ to 10^5^ bacteria). Moreover, no statistical differences are found between the viable bacterial counts from the sonication fluids of all *S. aureus*-challenged groups (*P* > 0.05). These findings could be explained by a consistent environmental situation in all cases, meaning comparable nutritional supply and amount of host defence mechanisms. The bacterial population would reach a similar steady state in all *S. aureus*-exposed groups, where the similar population size depended on the equal environmental conditions [31, 32]. The results of the present study therefore suggest that a stationary phase of bacterial population is reached during implant infection. This ultimate population size appears to be earlier reached on the implant surface than the peri-implant bone structures.

Consistent with publications on examination of human material the sonication of the implants used in this study to detach adhering bacteria was found to work reliably and to lead to consistent results. It has previously been described as precise, sensitive, and with a wide applicability [23]. Furthermore, complete and reproducible detachment of bacteria has been described for sonification [24] and as such it is currently gaining significance in the diagnosis of periprosthetic infection.

Probably related to the phenomenon of bacterial colonization rather than overt infection and also most likely due to way of implant insertion into the bone, that is, the introduction into the metaphysis while leaving the bone marrow intact, the radiological examination was uneventful for any of the tested animals. This finding is opposed to earlier findings on radiological signs of implant-related osteomyelitis induced by intramedullary implants [21]. Yet, results from conventional X-ray examinations are regarded as being variable and unspecific [33] and as such are inferior to microbiological and histological evaluation of implant-associated infections.

## 5. Conclusion

The results of the present study demonstrate the applicability of this novel animal model to reliably induce an implant-associated colonization and/or infection in the metaphysis of rats. The design of the conical hollow implant material allows for a deposition of defined bacterial loads without subsequent dilution or surgery technique-associated loss of substantial shares of the inoculum. We were able to confirm the findings of previous studies that a strong infection can be induced by comparatively small bacterial inocula since irrespective of the initial inoculum size a relatively constant population size is reached on the implant surface and in the neighbouring bone structures. Sonication of the implants is considered as a precise, sensitive, and widely applicable technique to suspend the bacterial biofilm from the implant surface. This novel animal model of implant-associated infection has the potential to become a standard for investigation of newly developed coated implant materials.

## Figures and Tables

**Table 1:** Bacteria counts inoculated during surgery.

	Ideal CFU	Experimentally determined CFU
Group I	0	0
Group II	10^3^	1,880 ± 219
Group III	10^4^	10,546 ± 7,986
Group IV	10^5^	124,881 ± 21,946
Group V	10^6^	670,000 ± 124,900

**Table 2:** Number of leukocytes (WBC), erythrocytes (RBC), and value of C-reactive protein (CRP) in blood samples.

CFU	Group I	Group II	Group III	Group IV	Group V
	(control)	(10^3^)	(10^4^)	(10^5^)	(10^6^)
WBC (×10 E9/l)—mean	7,81	5,63	7,54	6,7	11,66
WBC (×10 E9/l)—SD	3,43	1,27	3,7	1,73	6,55
RBC (×10 E12/l)—mean	7,65	7,08	7,12	7,28	7,68
RBC (×10 E12/l)—SD	0,29	0,22	0,73	0,84	0,41
CRP (mg/l)	<1	<1	<1	<1	<1

**Table:** (a)

	Number of initially seeded bacteria				
	PBS (neg. control)	10^3^	10^4^	10^5^	10^6^
Number of initially seeded bacteria					
PBS (neg. control)	—	0.000	0.000	0.000	0.024
10^3^	0.000	—	0.815	0.770	0.009
10^4^	0.000	0.815	—	0.806	0.002
10^5^	0.000	0.770	0.806	—	0.002
10^6^	0.024	0.009	0.002	0.002	—

**Table:** (b)

	Number of initially seeded bacteria				
	PBS (neg. control)	10^3^	10^4^	10^5^	10^6^
Number of initially seeded bacteria					
PBS (neg. control)	—	0.000	0.000	0.000	0.024
10^3^	0.000	—	0.187	0.123	0.064
10^4^	0.000	0.187	—	0.379	0.958
10^5^	0.000	0.123	0.379	—	0.824
10^6^	0.024	0.064	0.958	0.824	—
